# Understanding the Importance of Drag Coefficient Assessment for a Deeper Insight into the Hydrodynamic Profile of Swimmers

**DOI:** 10.5114/jhk/172492

**Published:** 2023-11-28

**Authors:** Jorge E. Morais, Daniel A. Marinho, Raul F. Bartolomeu, Tiago M. Barbosa

**Affiliations:** 1 Department of Sports Sciences, Instituto Politécnico de Bragança, Bragança, Portugal.; 2 Research Center in Sports, Health, and Human Development (CIDESD), Covilhã, Portugal.; 3 Department of Sports Sciences, University of Beira Interior, Covilhã, Portugal.; 4 Department of Sports Sciences, Polytechnic Institute of Guarda, Guarda, Portugal.

**Keywords:** swimming, hydrodynamics, technique, monitoring

## Abstract

The main objective of this study was to confirm that the passive drag coefficient is less dependent on swimming speed than the passive drag, Froude, and Reynolds numbers, even as swimming speed increases. The sample consisted of 12 young proficient non-competitive swimmers (seven males and five females: 20.4 ± 1.9 years). Passive drag was measured with a low-voltage isokinetic engine at 1.2, 1.4, 1.6 and 1.8 m/s. The frontal surface area was measured using digital photogrammetry. Passive drag showed significant differences with a strong effect size over the four towing speeds measured (F = 116.84, p < 0.001, η^2^ = 0.91) with a quadratic relationship with speed. The Froude and Reynolds numbers had similar trends, but with linear relationships. Conversely, the passive drag coefficient showed non-significant differences across the four towing speeds (F = 3.50, p = 0.062, η^2^ = 0.33). This strongly suggests that the passive drag coefficient should be the variable of choice for monitoring the hydrodynamic profile of swimmers rather than the absolute value of passive drag.

## Introduction

Swimming is highly dependent on the net balance between a thrust and hydrodynamic drag (Pendergast et al., 2005). The thrust is related to the ability to generate propulsive force to promote the forward displacement of the swimmer (Toussaint and Beek, 1992). On the other hand, hydrodynamic drag refers to the force that the water exerts on the swimmer, reducing his/her forward displacement (Pendergast et al., 2005). Therefore, swimming research has focused on understanding how to maximize the thrust and minimize hydrodynamic drag (Gatta et al., 2013; Morais et al., 2020).

Nevertheless, at constant speeds, the thrust is equal to hydrodynamic drag (Toussaint and Beek, 1992). Consequently, a reduction in drag can be used to achieve faster speeds for a given thrust production (Toussaint, 2011). Thus, the measurement and interpretation of hydrodynamic drag remains a key factor in improving swimming performance. Drag can be expressed by Newton’s equation as:


$$D=\frac{1}{2}\cdot v^{2}\cdot \rho \cdot S\cdot C_{D}\;\left(1\right)$$

where D is the drag force (in N), ρ is the density of water (in kg/m^3^), v is the swimming speed (in m/s), S is the projected frontal surface area (FSA) of the swimmers (in m^2^), and C_D_ is the coefficient of drag (changing according to shape, orientation, and Reynolds number). There are two types of drag: (i) active (D_A_) – the water resistance induced to a body while swimming (Kolmogorov and Duplishcheva, 1992), and (ii) passive (D_P_) – drag produced during the displacement of a towed body, i.e., without relative movement of the body segments in the aquatic environment (Pendergast et al., 2005).

In swimming events, swimmers spend most of their time performing the swim stroke, where D_A_ plays an important role (Arellano et al., 2022). Despite this, it was found that in 100-m sprint events, swimmers spent nearly a third of the race performing the start or turns (Morais et al., 2019). Thus, D_P_ remains of particular interest to researchers and coaches. In addition, despite the level of the competition, and if it is in a training or a teaching context, D_P_ is considered to be a good proxy for the hydrodynamic profile of swimmers (Chatard et al., 1990a).

In the past, when researchers wanted to gain insight into the hydrodynamic profile of swimmers, they often referred to the absolute value of D_A_ or D_P_ (Formosa et al., 2012; Gatta et al., 2013). However, it has been argued that the absolute value of hydrodynamic drag can give misleading interpretations and that the drag coefficient (C_D_) should be used (Morais et al., 2023). This is argued by the fact that the C_D_ is less dependent on swimming speed. This can be explained by measuring D_P_ and C_DP_ at different towing speeds and understand how this output changes with an increase in speed. Moreover, other proxies for the hydrodynamic profile of swimmers, such as the Froude number (F_r_), and Reynolds number (R_e_) are also used to understand the swimmers’ hydrodynamic profile. The F_r_ is considered to be a good proxy for wave making drag and is therefore also used as a hydrodynamic indicator (Kjendlie and Stallman, 2008). The R_e_ is another hydrodynamic indicator that is used to assess the state of the water flow around the swimmer (Silva et al., 2008).

Therefore, the main objective of this study was to confirm that the passive drag coefficient (C_DP_) is less dependent on swimming speed compared to other hydrodynamic variables, even as speed increases. Differences of D_P_, F_r_, R_e_, and C_DP_ over different towing speeds were tested. The D_P_–speed, F_r_–speed, R_e_–speed, and C_DP_–speed curves were also plotted. It was hypothesized that D_P_, F_r_, and R_e_ would present a greater difference between towing speeds than the C_DP_, and the remaining hydrodynamic variables would follow the same trend as D_P_. In addition, all hydrodynamic variables would have an exponential curve fitting.

## Methods

### Participants

The sample consisted of 12 young proficient swimmers (seven males and five females: 20.4 ± 1.9 years, 71.4 ± 8.9 kg of body mass, 1.73 ± 0.1 m of body height, 1.72 ± 0.1 m of the arm span, and a performance of 20.97 ± 3.50 s in a 25-m sprint test in front-crawl with an in-water push-off start) classified as Tier 2 athletes (McKay et al., 2022). For three months prior to data collection, swimmers participated in a twice weekly (three hours) swimming class program. Besides that, all swimmers had a swimming background with 4.1 ± 2.2 years of practice. As inclusion criterion, swimmers had to be able to master and maintain a streamlined position over the swimming pool length. This position is characterized by the upper limbs being fully extended above the head, one hand above the other, fingers also extended close together and head in a neutral position. Lower limbs must be fully extended in the projection of the trunk and feet in plantar flexion. Participants provided informed consent, and all procedures were in accordance with the Declaration of Helsinki for research involving human subjects. The Polytechnic Institute of Bragança Ethics Committee approved the research design (N.º 72/2022; approval date: 14 January 2022).

### Measurement of the Swimmer’s Height and the Frontal Surface Area (FSA)

Swimmers’ height (H, in cm) was measured as the distance from the vertex to the ground (with swimmers in the orthostatic position) using a digital stadiometer (SECA, 242, Hamburg, Germany).

For the FSA measurement, each swimmer was photographed with a digital camera (a6000, Sony, Tokyo, Japan) in the transverse plane from above (Morais et al., 2011). They were on land, in the upright and hydrodynamic position. This position is characterized by arms fully extended above the head, one hand on top of the other, fingers also extended close together, and the head in a neutral position. Swimmers wore a regular textile swimsuit, a cap, and goggles. The FSA was measured from the digital photograph of the swimmer using dedicated software (Udruler, AVPSoft, USA). The procedures for the FSA measurement were based on: (i) scale calibration; (ii) manual digitization of the FSA; and (iii) output and recording of the FSA value (Morais et al., 2011).

### Measurement of Passive Drag (DP) and the Passive Drag Coefficient (CDP)

For the D_P_ measurement, swimmers were asked to adopt the same streamlined and hydrodynamic position as for the FSA measurement. They were also instructed to hold on to the wire and to hold their breath after a maximal inhalation (Gatta et al., 2013). The lower limbs were passively lifted using a standard eight-shaped pull-buoy (23 x 13 x 8 cm; Golfinho, Portugal). Swimmers were asked to grasp a non-elastic wire attached to a low-voltage isokinetic engine (Ben Hur, ApLAb, Rome, Italy) placed at the edge of the swimming pool (Gatta et al., 2013). The engine rolled the wire at a constant speed across the 25-m length of the swimming pool. Four towing speeds were used: 1.2 m/s, 1.4 m/s, 1.6 m/s, and 1.8 m/s. The engine was calibrated according to the manufacturer’s instructions prior to each experimental session. A GoPro Hero 7 video camera (at a sampling rate of 60 Hz; GoPro, San Mateo, CA, USA) was synchronized to the software to record the swimmers’ performance in the sagittal plane. The data were then downloaded to a PC and processed using signal processing software (AcqKnowledge v. 3.9.0, Biopac Systems, Santa Barbara, USA). The average force (i.e., D_P_) between the 10^th^ and the 20^th^ m from the start was used for analysis, as suggested by others (Gatta et al., 2013; Zamparo et al., 2009).

The C_DP_ for each towing speed was calculated as follows:


$$C_{DP}=\frac{D_{P}}{0.5\cdot \rho \cdot v^{2}\cdot FSA}\;\left(2\right)$$

where C_DP_ is the passive drag coefficient (dimensionless), D_P_ is passive drag (N), ρ is the density of water (assumed to be 997 kg/m^3^), v is the swimming speed (m/s), and the FSA is the previously measured frontal surface area (m^2^).

The F_r_ was calculated as:


$$F_{r}=\frac{v}{\sqrt{g\cdot H}}\;\left(3\right)$$

where F_r_ is the Froude number (dimensionless), v is the swimming speed (in m/s), g is the gravitational acceleration (which is 9.81 m/s^2^), and His height of the swimmer (in m). The R_e_ was calculated as:


$$R_{e}=\frac{v\cdot H}{\upsilon }\;\left(4\right)$$

where R_e_ is the Reynolds number (dimensionless), v is the swimming speed (in m/s), H is height of the swimmer (in m), and υ is the kinematic viscosity of water (which is 8.97 × 10^−7^ m^2^/s at 26°C).

### Statistical Analysis

The Kolmogorov-Smirnov and the Levene’s tests were used to assess the normality and homoscedasticity, respectively. Descriptive statistics were calculated as mean plus one standard deviation (SD). The magnitude of the difference between speeds for all variables was calculated using the coefficient of variation (CV = one standard deviation / mean * 100, in %).

One-way repeated measures ANOVA was used to test for within-subject differences in the speeds used (*p* < 0.05). The effect size index (eta squared – η^2^) was calculated and interpreted as: (i) no effect if 0 < η^2^ < 0.04; (ii) minimal if 0.04 < η^2^ < 0.25; (iii) moderate if 0.25 < η^2^ < 0.64; and (iv) strong if η^2^ > 0.64 (Ferguson, 2009). If necessary, the Bonferroni post-hoc correction would be used to test for pairwise differences. Cohen’s *d* was used to estimate the standardized effect sizes, which were considered to be: (i) trivial if 0 ≤ *d* < 0.20; (ii) small if 0.20 ≤ *d* < 0.60; (iii) moderate if 0.60 ≤ *d* < 1.20; (iv) large if 1.20 ≤ *d* < 2.00; (v) very large if 2.00 ≤ *d* < 4.00; (vi) nearly distinct if *d* ≥ 4.00 (Hopkins, 2002).

Curve fitting was used to model the D_P_–speed, F_r_–speed, R_e_–speed, and the C_D_–speed by assigning the “best fit” function over the measured swim speeds. For this, linear, quadratic, and cubic fits were tested. Trendline, 95CI, and standard error of estimation (SEE) were calculated. The SEE was used as an indicator of goodness-of-fit to compare the models (i.e., linear, quadratic, and cubic) (Siegel, 2016). It indicates how accurate the model predictions are using the units of the dependent variable (i.e., it indicates how far the data points are from the regression line on average). Lower values of the SEE indicate that the distances between the data points and the fitted values are smaller (i.e., best fit). All statistical analyses were performed using IBM SPSS (version 26; SPSS Inc, Chicago, IL).

## Results

Swimmers had a mean FSA of 788.72 ± 109.93 cm^2^ (males: 819.49 ± 134.04 cm^2^; females: 745.64 ± 47.99 cm^2^). All hydrodynamic variables increased as the towing speed increased (Table 1). The largest CV was found between towing speeds of 1.2 m/s and 1.8 m/s with 45.25%, 20.00%, 150.00%, and 8.96%, for D_P_, F_r_, R_e_, and C_DP_, respectively (Table 2).

**Table 1:** Descriptive statistics by towing speed and difference analysis.

	Mean ± 1SD				ANOVA	
	1.2 m/s	1.4 m/s	1.6 m/s	1.8 m/s	F-ratio (*p*-value)	η^2^
**D_P_ [N]**	34.12 ± 8.04	49.08 ± 10.33	63.99 ± 10.78	90.52 ± 11.43	116.84 (< 0.001)	0.91
**F_r_ [dimensionless]**	0.29 ± 0.01	0.34 ± 0.01	0.39 ± 0.01	0.44 ± 0.01	5359.51 (< 0.001)	1.00
**R_e_ (×10^6^) [dimensionless]**	2.31 ± 0.12	2.69 ± 0.15	3.08 ± 0.17	3.46 ± 0.19	4045.13 (< 0.001)	0.98
**C_DP_ [dimensionless]**	0.61 ± 0.13	0.64 ± 0.12	0.65 ± 0.12	0.73 ± 0.16	3.50 (0.062)	0.33

D_P_ – passive drag; F_r_ – Froude number; R_e_ – Reynolds number; C_DP_ – passive drag coefficient; η^2^ – eta squared (effect size index).

**Table 2:** Pairwise comparison between towing speeds.

		1.2 vs. 1.4 m/s	1.2 vs. 1.6 m/s	1.2 vs. 1.8 m/s	1.4 vs. 1.6 m/s	1.4 vs. 1.8 m/s	1.6 vs. 1.8 m/s
D_P_ [dim.]	MD (*p*-value)	14.96 (< 0.001)	29.87 (< 0.001)	56.40 (< 0.001)	14.91 (< 0.001)	41.44 (< 0.001)	26.53 (< 0.001)
	95CI	7.69 to 22.23	23.40 to 36.35	42.61 to 70.20	7.78 to 22.04	29.07 to 53.81	15.47 to 37.59
	d [descriptor]	1.62 [large]	3.13 [very large]	5.71 [nearly distinct]	1.41 [large]	3.80 [very large]	2.39 [very large]
	CV	17.98	30.45	45.25	13.19	29.68	17.17
F_r_ [dim.]	MD (*p*-value)	−0.048 (< 0.001)	−0.097 (< 0.001)	−0.146 (< 0.001)	−0.049 (< 0.001)	−0.097 (< 0.001)	−0.048 (< 0.001)
	95CI	−0.052 to −0.045	−0.102 to −0.093	−0.151 to −0.141	−0.052 to −0.046	−0.102 to −0.093	−0.052 to −0.045
	d [descriptor]	5.00 [nearly distinct]	10.00 [nearly distinct]	15.00 [nearly distinct]	5.00 [nearly distinct]	10.00 [nearly distinct]	5.00 [nearly distinct]
	CV	7.69	14.29	20.00	6.67	12.50	5.88
R_e_ (×10^6^) [dim.]	MD (*p*-value)	−0.385 (< 0.001)	−0.769 (< 0.001)	−1.154 (< 0.001)	−0.385 (< 0.001)	−0.769 (< 0.001)	−0.385 (< 0.001)
	95CI	−0.404 to −0.365	−0.808 to −0.730	−1.212 to −1.096	−0.404 to −0.365	−0.808 to −0.730	−0.404 to −0.365
	d [descriptor]	2.80 [very large]	5.23 [nearly distinct]	7.24 [nearly distinct]	2.43 [very large]	4.50 [nearly distinct]	2.11 [very large]
	CV	116.67	133.33	150.00	114.29	128.57	112.50
C_DP_ [dim.]	CV	2.40	3.17	8.96	0.78	6.57	5.80

dim – dimensionless; MD – mean difference; 95CI – 95% confidence intervals; d – Cohen’s effect size; CV – coefficient of variation; D_P_ – passive drag; F_r_ – Froude number; R_e_ – Reynolds number; C_DP_ – passive drag coefficient.

D_P_ showed significant differences with a strong effect size over the four towing speeds measured (F = 116.84, *p* < 0.001, η^2^ = 0.91). The highest value was obtained at the fastest towing speed (90.52 ± 11.43 N at 1.8 m/s) (Table 1). Post-hoc correction revealed that the largest and most meaningful difference (with a nearly distinct effect size) was observed between 1.2 and 1.8 m/s towing speeds (mean difference = 56.40 N, 95CI = 42.61 to 70.20, *d* = 5.71) (Table 2). The F_r_ and R_e_ showed a similar trend. Conversely, the C_DP_ showed non-significant differences across the four towing speeds, but with a moderate effect size (F = 3.50, *p* = 0.062, η^2^ = 0.33).

**Figure 1:**
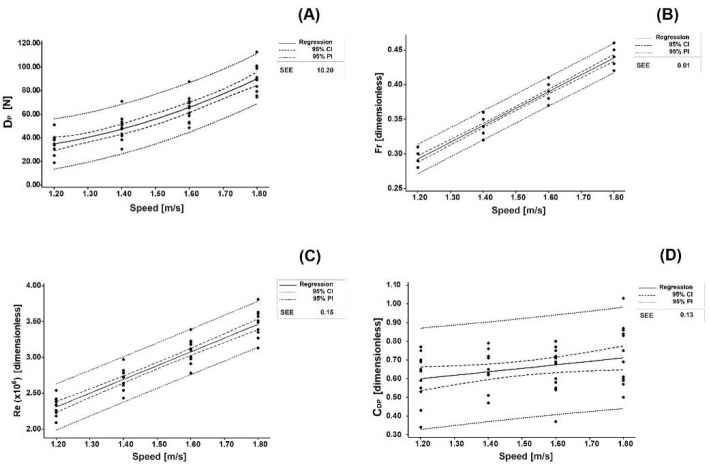
Curve fitting of D_P_ (Panel A), F_r_ (Panel B), R_e_ (Panel C), and C_DP_ (Panel D) over the four towing speeds. In all panels, 95CI stands for 95% confidence intervals, 95PI stands for 95% prediction intervals, and SEE stands for standard error of estimation.


The curve fitting for the D_P_–speed over the four towing speeds showed a quadratic relationship (SEE = 10.20) (Figure 1 – Panel A):

$$D_{P}=80.37-124.80\;\times v+72.29\times v^{2}\;\left(5\right)$$

where D_P_ is passive drag (in N) and v is the swimming speed (in m/s). The F_r_–speed showed a linear relationship (SEE = 0.01) (Figure 1 – Panel B):


$$F_{r}=0.00042+0.2433\;\times v\;\left(6\right)$$

where F_r_ is the Froude number (dimensionless) and v is the swimming speed (in m/s). The R_e_–speed showed a linear relationship (SEE = 0.15) (Figure 1 – Panel C):


$$R_{e}=0.0029+1.921\;\times v\;\left(7\right)$$

where R_e_ is the Reynolds number (dimensionless) and v is the swimming speed (in m/s). The C_D_–speed showed a linear relationship (SEE = 0.13) (Figure 1 – Panel D):


$$C_{DP}=0.3758+0.1862\;\times v\;\left(8\right)$$

where C_DP_ is the passive drag coefficient (dimensionless) and v is the swimming speed (in m/s).

## Discussion

The purpose of this study was to confirm that the C_DP_ is less dependent on swimming speed compared to other hydrodynamic variables, even when swimming speed increases. The difference of D_P_, F_r_, R_e_, and C_DP_ over different towing speeds was measured, and the D_P_–speed, F_r_–speed, R_e_–speed, and C_DP_–speed curves were modeled. The main results indicated that D_P_, F_r_, and R_e_ showed significant differences between the towing speeds. D_P_ showed a quadratic relationship with towing speed, while F_r_ and R_e_ showed a linear relationship. Conversely, the C_DP_ did not show significant differences over the four towing speeds and a linear relationship was found.

The present data showed that D_P_ increased significantly and meaningfully as the speed increased and with a quadratic relationship. This is because drag depends on the square of speed, thus an increase in swimming speed causes an exponential increase in drag (Toussaint and Beek, 1992). Therefore, to understand if swimmers are improving their hydrodynamic profile (i.e., their resistance to water), they must be measured at the same speed. Consequently, there are two variables that can be responsible for such an improvement: FSA and C_D_. For swimmers who have already reached their growth development and have already mastered the streamlined position, the FSA may not likely change in a meaningful way. Thus, the C_D_ (that changes according to the shape, orientation, and Reynolds number) will be the variable responsible for such improvement. Considering young swimmers, they tend to meaningfully increase their swimming speed due to growth and technical development (Kuberski et al., 2022; Morais et al., 2013). However, drag also increases with speed. Thus, it becomes more difficult to interpret such results, i.e., how to justify the technical and hypothetical hydrodynamic improvement when drag increases exponentially. The F_r_ and R_e_, variables related to hydrodynamics, showed the same trend as D_P_ (but with a linear relationship), where significant differences were also found between the speeds. This suggests that these variables are highly speed dependent.

Most research on drag in swimming refers to its absolute value for both active (Formosa et al., 2012; Neiva et al., 2021) and passive conditions (Gatta et al., 2016; Narita et al., 2017). That is, researchers use the absolute value of drag to understand its relationship with swimming performance. For example, two of the most cited articles on swimmers’ D_A_ (Toussaint et al., 2004) and D_P_ (Chatard et al., 1990b) referred only to the absolute value. Although there are more recent articles reporting the C_D_ for both active and passive conditions (Barbosa et al., 2013), most articles on this topic still exclude the C_D_ from the results. In contrast, C_DP_ did not differ significantly between towing speeds (with a moderate effect size), had a linear relationship, and was also the variable with the lowest CV between towing speeds. This indicates that the C_DP_ is the hydrodynamic indicator that is less sensitive or dependent on speed. Indeed, in terrestrial sports where aerodynamics play an important role, such as cycling (Blocken et al., 2018) or speed skating (Brownlie, 2021), the C_D_ is the output used to analyze the athletes’ aerodynamics. Therefore, the present findings support the importance of the C_D_, specifically the C_DP_, and why it should be the output to be used when aiming to understand the hydrodynamic profile of swimmers. This gives coaches and swimmers a better understanding of the swimmer’s hydrodynamic profile. Moreover, it shows that the staff responsible for the swimmers’ research and development must rely on the C_DP_ rather than the absolute value of drag when referring to the swimmers’ hydrodynamics.

The main limitation of this study includes the level of the swimmers recruited. One could argue about the level of mastering the streamlined position to evaluate the hydrodynamic drag. However, these were proficient swimmers, and the streamlined position quality was evaluated as an inclusion criterion. Moreover, future studies should be conducted to understand this phenomenon in active conditions.

## Conclusions

Of the four hydrodynamic variables measured, the C_DP_ was the one that showed the least dependence on swimming speed. This suggests that this variable should be used to understand the hydrodynamic profile of swimmers.
